# CD26/DPP4 Cell-Surface Expression in Bat Cells Correlates with Bat Cell Susceptibility to Middle East Respiratory Syndrome Coronavirus (MERS-CoV) Infection and Evolution of Persistent Infection

**DOI:** 10.1371/journal.pone.0112060

**Published:** 2014-11-19

**Authors:** Yíngyún Caì, Shuǐqìng Yú, Elena N. Postnikova, Steven Mazur, John G. Bernbaum, Robin Burk, Téngfēi Zhāng, Sheli R. Radoshitzky, Marcel A. Müller, Ingo Jordan, Laura Bollinger, Lisa E. Hensley, Peter B. Jahrling, Jens H. Kuhn

**Affiliations:** 1 Integrated Research Facility at Fort Detrick, National Institute of Allergy and Infectious Diseases, National Institutes of Health, Fort Detrick, Frederick, Maryland, United States of America; 2 United States Army Medical Research Institute of Infectious Diseases, Fort Detrick, Frederick, Maryland, United States of America; 3 Institute of Virology, University of Bonn Medical Centre, Bonn, Germany; 4 ProBioGen AG, Berlin, Germany; Faculty of Biochemistry Biophysics and Biotechnology, Jagiellonian University, Poland

## Abstract

Middle East respiratory syndrome coronavirus (MERS-CoV) is a recently isolated betacoronavirus identified as the etiologic agent of a frequently fatal disease in Western Asia, Middle East respiratory syndrome. Attempts to identify the natural reservoirs of MERS-CoV have focused in part on dromedaries. Bats are also suspected to be reservoirs based on frequent detection of other betacoronaviruses in these mammals. For this study, ten distinct cell lines derived from bats of divergent species were exposed to MERS-CoV. Plaque assays, immunofluorescence assays, and transmission electron microscopy confirmed that six bat cell lines can be productively infected. We found that the susceptibility or resistance of these bat cell lines directly correlates with the presence or absence of cell surface-expressed CD26/DPP4, the functional human receptor for MERS-CoV. Human anti-CD26/DPP4 antibodies inhibited infection of susceptible bat cells in a dose-dependent manner. Overexpression of human CD26/DPP4 receptor conferred MERS-CoV susceptibility to resistant bat cell lines. Finally, sequential passage of MERS-CoV in permissive bat cells established persistent infection with concomitant downregulation of CD26/DPP4 surface expression. Together, these results imply that bats indeed could be among the MERS-CoV host spectrum, and that cellular restriction of MERS-CoV is determined by CD26/DPP4 expression rather than by downstream restriction factors.

## Introduction

In 2012, a novel human coronavirus causing frequently fatal disease emerged in Western Asia [1] and was named “Middle East respiratory syndrome coronavirus (MERS-CoV)” [2]. As of June 11, 2014, MERS-CoV caused 699 laboratory-confirmed human infections in 21 countries, including 209 deaths (proportion of fatal cases ≈29.9%) [3]. Increasing evidence points to dromedaries (*Camelus dromedarius*) as an intermediate reservoir contributing to the emergence of Middle East respiratory syndrome (MERS) in humans. Several seroepidemiology studies have found MERS-CoV-neutralizing antibodies in dromedaries from Egypt, Jordan, Oman, and Saudi Arabia [4]–[9]. More recently, coronaviral genomes detected in nasal swabs obtained from dromedaries proved to be identical to genomes of human MERS-CoV isolates [4], [5], [9]. One such genome was detected in a patient who had been caring for a sick dromedary and directly from that animal [10]. In addition, MERS-CoV was directly isolated from a dromedary in Qatar [11].

The source of dromedary MERS-CoV infection remains to be elucidated, but it is not unlikely that they serve only as intermediary hosts [12]. Bats have been proposed as additional MERS-CoV hosts. This hypothesis is based on the fact that several betacoronaviruses related to MERS-CoV (e.g., severe acute respiratory syndrome-like coronaviruses, Tylonycteris bat coronavirus HKU4, Pipistrellus bat coronavirus HKU5) are known to infect bats in Africa, Europe, and Asia [1], [13]–[16]. In addition, MERS-CoV genome fragments encoding parts of the RNA-dependent RNA polymerase were detected in one Egyptian tomb bat (*Taphozous perforates*) living close to a MERS-CoV-infected patient [14]. Finally, a novel coronavirus (NeoCoV) closely related to MERS-CoV was discovered in cape serotines (*Neoromicia capensis*) in South Africa [12]. Therefore, bats could possibly maintain MERS-CoV in nature and may occasionally infect dromedaries and thereby may infect humans similar to the bats-horse-human or bats-pig-human transmission cycle observed for henipaviruses [17], [18].

Recently, CD26, also known as dipeptidyl peptidase 4 (DPP4) was identified as the human MERS-CoV cell entry receptor [19] and also as a receptor for Tylonycteris bat coronavirus HKU4 [20], [21]. CD26/DPP4 receptor is conserved among different mammals (e.g., bats, dromedaries, humans), and the possibly broad species tropism of MERS-CoV may partly be the result of this conservation [19], [22].

To further evaluate the hypothesis that bats may be implicated in transmission of the virus, we inoculated ten cell lines from phylogenetically diverse bats living in geographically distinct areas with MERS-CoV. Six bat cell lines were productively infected. The susceptibility or resistance of the ten cell lines to MERS-CoV infection directly correlated with the absence or presence of naturally expressed CD26/DPP4 on the cells surface. Anti-human CD26/DPP4 antibodies reduced MERS-CoV yield in susceptible bat cell cultures in a dose-dependent manner. Similar to other studies using MERS-CoV-resistant (non-bat) cell lines transfected with CD26/DPP4 [19], expression of human CD26/DPP4 in resistant bat cells rendered these cell lines susceptible to infection. Finally, we demonstrate that persistent MERS-CoV infections can be established in permissive bat cell lines after sequential virus passage, leading to downregulation of natural CD26/DPP4 cell-surface expression. Together, our data indicate that the host cell tropism of MERS-CoV may largely depend on the expression of suitable CD26/DPP4 orthologs, and that bats cannot be excluded as MERS-CoV reservoirs at this point in time.

## Materials and Methods

### Cell lines

Huh-7 (a kind gift from Hideki Ebihara, Rocky Mountain Laboratory, Hamilton, MT), Vero E6 (ATCC, #CRL-1568, Manassas, VA), and Vero (ATCC, #CCL-81) cells were grown in Dulbecco's modified Eagle's medium (DMEM) supplemented with 10% heat-inactivated fetal bovine serum (FBS, Sigma-Aldrich, St. Louis, MO). The bat cell lines used in this study are described in Table 1. R05T, R06E, and HypNi/1.1 cell lines were grown in DMEM/F-12 (Lonza, Walkersville, MD) supplemented with 10% FBS. All others bat cell lines were maintained in DMEM supplemented with 10% FBS. All cells were incubated at 37°C in a humidified 5% CO_2_ atmosphere.

**Table 1:** Origin of tested bat cell lines.

Bat cell line [reference]	Origin	Type of bat	Geographic distribution of bat (according to http://www.iucnredlist.org)
EidNi/41.3 [35]	African straw-colored fruit bat (*Eidolon helvum*) adult kidney	pteropid (frugivorous)	Angola; Benin; Botswana; Burkina Faso; Burundi; Cameroon; Central African Republic; Chad; Côte d'Ivoire; Democratic Republic of the Congo; Equatorial Guinea; Ethiopia; Gabon; Gambia; Ghana; Guinea; Guinea-Bissau; Kenya; Lesotho; Liberia; Malawi; Mali; Mauritania; Mozambique; Namibia; Niger; Nigeria; Republic of Congo; Rwanda; Sao Tomé and Principe; **Saudi Arabia^a^**; Senegal; Sierra Leone; South Africa; South Sudan; Sudan; Swaziland; Tanzania; Togo; Uganda; **Yemen**; Zambia; Zimbabwe
EpoNi/22.1 [36]	Büttikofer's epauletted fruit bat (*Epomops buettikoferi*) adult kidney	pteropid (frugivorous)	Côte d'Ivoire; Ghana; Guinea; Guinea-Bissau; Liberia; Nigeria; Senegal; Sierra Leone
HypLu/45.1 [36]	hammer-headed fruit bat (*Hypsignathus monstrosus*) fetal lung	pteropid (frugivorous)	Angola; Benin; Burkina Faso; Cameroon; Central African Republic; Côte d'Ivoire; Democratic Republic of the Congo; Equatorial Guinea; Ethiopia; Gabon; Ghana; Guinea; Guinea-Bissau; Kenya; Liberia; Nigeria; Republic of the Congo; Sierra Leone; South Sudan; Togo; Uganda
HypNi/1.1 [36]	hammer-headed fruit bat (*Hypsignathus monstrosus*) fetal kidney	pteropid (frugivorous)	See HypLu/45.1
PESU-B5L [37]	eastern pipistrelle (*Pipistrellus subflavus*) adult lung	vespertilionid (insectivorous)	Belize; Canada; Guatemala; Honduras; Mexico; United States
R05T [38]	Egyptian rousette (*Rousettus aegyptiacus*) embryo	pteropid (frugivorous)	Angola; Burundi; Cameroon; Côte d'Ivoire; Cyprus; Democratic Republic of the Congo; **Egypt**; Equatorial Guinea; Eritrea; Ethiopia; Gabon; Gambia; Ghana; Guinea; **Iran**; Israel; **Jordan**; Kenya; **Lebanon**; Lesotho; Liberia; Libya; Malawi; Mozambique; Nigeria; **Oman**; Pakistan; Republic of the Congo; Rwanda; Sao Tomé and Principe; **Saudi Arabia**; Senegal; Sierra Leone; South Africa; South Sudan; Sudan; Syrian Arab Republic; Tanzania; Togo; Turkey; Uganda; **United Arab Emirates**; **Yemen**; Zambia; Zimbabwe
R06E [38]	Egyptian rousette (*Rousettus aegyptiacus*) embryo	pteropid (frugivorous)	See R05T
RoNi/7.1 [36]	Egyptian rousette (*Rousettus aegyptiacus*) adult kidney	pteropid (frugivorous)	See R05T
RoNi/7.2 - subclone of RoNi/7 used in [39]	Egyptian rousette (*Rousettus aegyptiacus*) adult kidney	pteropid (frugivorous)	See R05T
Tb1Lu [ATCC (#CCL-88)]	Brazilian free-tailed bat (*Tadarida brasiliensis*) adult lung	molossid (insectivorous)	Anguilla; Antigua and Barbuda; Argentina; Aruba; Barbados; Bolivia; Bonaire, Sint Eustatius and Saba; Brazil; Chile; Colombia; Costa Rica; Cuba; Dominica; Dominican Republic; Ecuador; El Salvador; French Guiana; Grenada; Guadeloupe; Guatemala; Guyana; Haiti; Honduras; Jamaica; Martinique; Mexico; Montserrat; Panama; Peru; Puerto Rico; Saint Kitts and Nevis; Saint Lucia; Saint Martin (French part); Saint Vincent and the Grenadines; Sint Maarten (Dutch part); Suriname; Trinidad and Tobago; United States; Venezuela; Virgin Islands

a Western Asian and Northern African countries in which MERS cases have been documented are printed in bold.

### Virus propagation

Middle East respiratory syndrome coronavirus isolate HCoV-EMC/2012 (MERS-CoV/EMC) was kindly provided by the Department of Viroscience Lab, Erasmus University Medical Center, Rotterdam, NL. MERS-CoV isolate Hu/Jordan-N3/2012 (MERS-CoV/Jor) was kindly provided by Drs. Kanta Subbarao (National Institutes of Health, Bethesda, MD) and Gabriel Defang (Naval Medical Research Unit-3, Cairo, EG). Both viruses were propagated in Vero E6 cells at a multiplicity of infection (MOI) of 0.01 in DMEM supplemented with 2% FBS. The viruses were titrated on Vero cells by plaque assay.

### Infection of bat cell lines

Bat cell lines were seeded in collagen-coated 24-well plates (Becton Dickinson Labware, Bedford, MA) at 2×10^5^ cells/well. One day later, media were removed, and cells were washed once with DMEM without FBS (0% DMEM). Cells were then exposed to MERS-CoV/EMC or MERS-CoV/Jor at an MOI of 1. After 1 h of incubation at 37°C, viral inocula were removed and cells were washed once with 0% DMEM and then supplemented with DMEM containing 2% FBS (2% DMEM). At 1, 3, or 5 days post-exposure, supernatants were harvested and cleared of cellular debris by centrifugation.

### Plaque assay

MERS-CoV particle yields were quantified by plaque assay [23]. Briefly, confluent monolayers of Vero cells in 6-well plates were exposed to serial dilutions of MERS-CoV, incubated at 37°C for 1 h under gentle rocking every 15 min, followed by removal of inocula and addition of a 0.8% tragacanth overlay (Sigma-Aldrich, St. Louis, MO). Infected cells were then incubated at 37°C for 72 h. The tragacanth overlay was removed, and the cells were stained with 2% crystal violet (Sigma-Aldrich) in 10% neutral buffered formalin (NBF, Fisher Scientific, Kalamazoo, MI). Plaques were enumerated manually.

### CD26/DPP4 antibody inhibition assay

RoNi/7.1 or Huh-7 cells were incubated with different concentrations (0, 1.25, 2.5, 5, 10, 20 µg/ml) of goat anti-human CD26/DPP4 antibody (R&D Systems, Minneapolis, MN) or control goat IgG antibody at 37°C for 1 h. Antibody-treated cells were exposed to MERS-CoV/EMC at an MOI of 1 at 37°C for 1 h in the presence of antibodies. Virus-antibody inocula were then removed, cells were washed in 0% DMEM, fresh DMEM (2% FBS) was added, supernatants were harvested 24 h post-exposure, and viral yields were determined by plaque assay. At the same time, plates were fixed with 10% NBF and then stained with rabbit polyclonal anti-MERS-CoV spike protein antibody (Sino Biological, Beijing, China) followed by secondary Alexa Fluor 488-conjugated goat anti-rabbit IgG antibody (Life Technologies, Carlsbad, CA). Hoechst 33342 dye was used to stain nuclei. The percentage of infected cells was measured and analyzed using the Operetta high content imaging system (PerkinElmer Waltham, MA) and analysis software (Harmony 3.1).

### Flow cytometry

Bat cells were washed with phosphate-buffered saline (PBS) and then dissociated with cell dissociation buffer (Life Technologies). Cells were spun down, washed, and resuspended in 4% paraformaldehyde for fixation. Cells were stained with goat anti-human CD26/DPP4 antibody followed by Alex Fluor 488-conjugated rabbit anti-goat IgG antibody. As a control, the cells were stained with the same concentration of isotype control goat IgG antibody followed by the same secondary antibody. Samples were collected using an LSR Fortessa flow cytometer (BD Biosciences, San Jose, CA). FlowJo software version 9.7.5 (TreeStar, Ashland, OR) was used to analyze the data.

### Transmission electron microscopy

Confluent bat cells were inoculated with MERS-CoV/EMC at an MOI of 1 for 1 h at 37°C. After viral inocula were removed, cells were washed once with 0% DMEM and then supplemented with 2% DMEM. Media were removed 24 h later, and electron microscopy grade fixative, 2.5% glutaraldehyde (E.M. Sciences, Warrington, PA) in Millonig's sodium phosphate buffer (Tousimis Research, Rockville, MD), was added directly to the dishes. After 10 min, bat cells were scraped off the dishes with a cell scraper, collected into 15-ml tubes, and immediately centrifuged at 500×*g* for 20 min. To complete fixation, cells were kept in fixative for 24 h at 4°C and were post-fixed in 1% osmium tetroxide (Electron Microscopy Sciences, Hatfield, PA). Post-fixed cells were stained *en bloc* with 2% uranyl acetate, dehydrated in a series of graded ethanols, and infiltrated and embedded in Spurr plastic resin (Electron Microscopy Sciences). A Leica EM UC7 ultramicrotome (Leica Microsystems, Buffalo Grove, IL) was used to section the embedded blocks into ultra-thin sections (60–80 nm). These sections were collected, mounted on 200-mesh copper grids (Electron Microscopy Sciences), and contrasted with Reynold's lead citrate. A FEI G2 Tecnai transmission electron microscope (FEI, Hillsboro, OR), operating at 80 kV, was used to examine and image the grids.

### CD26 overexpression experiments

MERS-CoV-resistant PESU-B5L, R05T, R06E, or Tb1Lu or MERS-CoV-susceptible EidNi/41.3, EpoNi/22.1, HypLu/45.1, HypNi/1.1, RoNi/7.1, RoNi/7.2, or Vero E6 cells were transfected with a plasmid expressing human CD26/DPP4 (pCMV-xL-hDPP4, Origene Technologies, Rockville, MD) or control plasmid pcDNA3.1+ (Life Technologies) by Effectene (Qiagen, Frederick, MD) or Lipofectamine 3000 (Life Technologies) according to the manufacturer's instruction. At 24 h or 48 h post transfection, cells were washed once with 0% DMEM and then inoculated with MERS-CoV/EMC at an MOI of 3. Bat cells were incubated at 37°C for 1 h with gently rocking of the plates every 15 min. At 1 h after exposure, cells were washed twice with 0% DMEM, and 0.5 ml of 2% DMEM was added. At 24 h post-exposure, supernatants were harvested for virus yield determination. Plates were fixed with 10% NBF. Plates were stained with goat anti-human CD26/DPP4 followed by Alexa Fluor 594-conjugated donkey anti-goat IgG antibody and/or polyclonal rabbit anti-MERS-CoV spike protein antibody followed by Alex Fluor 488-conjugated chicken anti-rabbit IgG antibody (Life Technologies). Images were acquired using the Operetta high content imaging system.

### Establishment of persistent MERS-CoV infection

EidNi/41.3, EpoNi/22.1, HypLu/45.1, HypNi/1.1, RoNi/7.1, RoNi/7.2, or Vero E6 cells in 75 cm^2^ flasks were infected with MERS-CoV/EMC or MERS-CoV/Jor at an MOI of 1. After 7 days, supernatants were harvested for virus yield analysis by plaque assay, and the cells were subcultured at a 1∶10 dilution in new flasks. Subsequently, the infected cells were passaged at a 1∶10 dilution weekly for a total of nine passages. From each passage, supernatants were harvested, and virus yields were determined by plaque assay.

### Western blot analysis

EidNi/41.3 cells (non-infected or persistently infected with MERS-CoV, day 63) were washed with PBS and lysed in cell lysis buffer (Cell Signaling, Danvers, MA) according to the manufacturer's instruction. Equivalent amounts of total cellular lysates were resolved in 4% to 12% bis-tris gradient gels (Life Technologies) and then dry-transferred to polyvinylidene difluoride (PVDF) membranes (Life Technologies) by using the iBlot gel transfer system (Life Technologies). After blocking in 5% nonfat milk powder in PBS with 0.1% Tween (Sigma-Aldrich), membranes were incubated overnight with goat anti-human CD26/DPP4 antibody (1∶500) or anti β-actin antibody (1∶500, Abcam, Cambridge, MA), followed by incubation with appropriate horseradish peroxidase-conjugated secondary antibodies (Sigma-Aldrich). Signals were detected by SuperSignal West Femto chemiluminescent substrate (Thermo Fisher Scientific, Rockford, IL), and images were acquired using a Syngene G: Box Chemiluminescene imaging system (Syngene, Frederick, MD).

## Results

### Six of ten tested bat cell lines are susceptible to MERS-CoV infection

**Figure 1:**
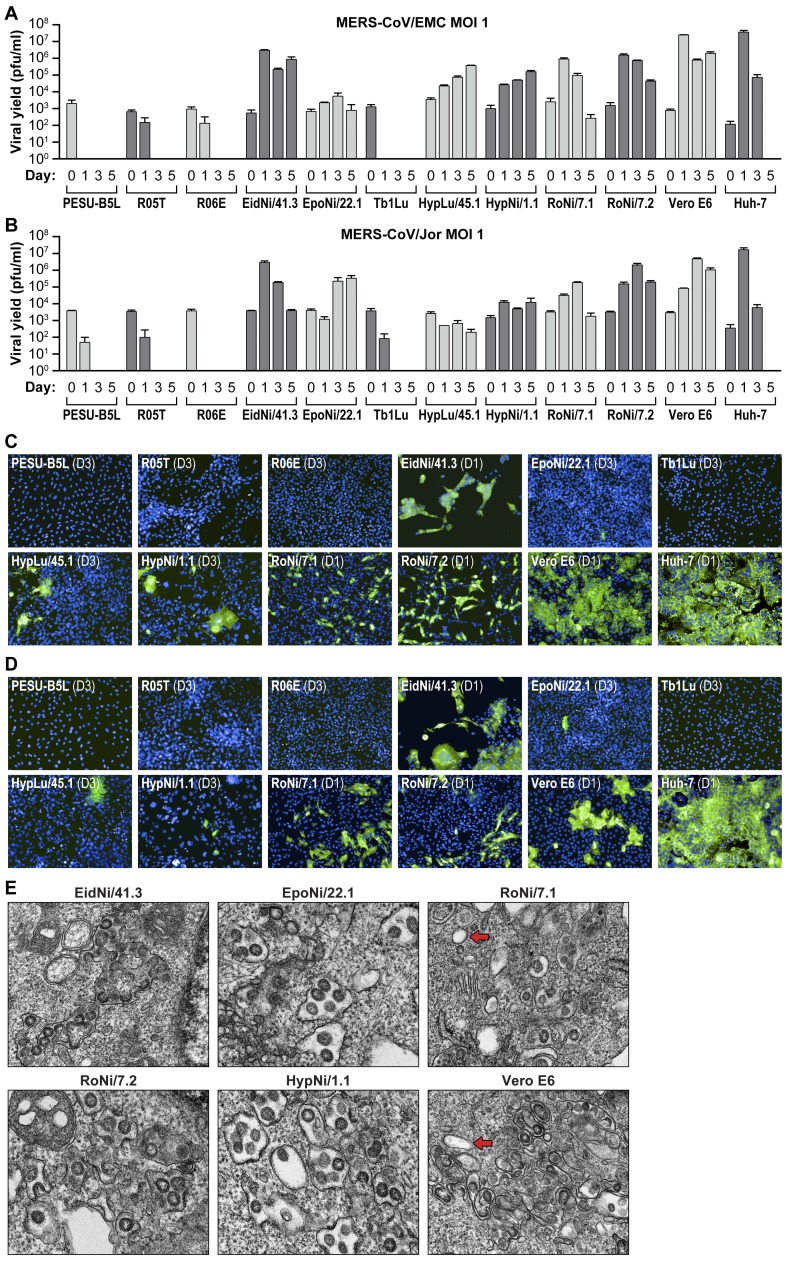
Six of ten tested bat cell lines are susceptible to MERS-CoV infection. (A and B) Ten different bat cell lines were exposed to MERS-CoV/EMC (A) or MERS-CoV/Jor (B) at an MOI of 1. Supernatants were harvested at days 0, 1, 3, and 5 after virus exposure, and virus yields were determined by plaque assay on Vero cells. Error bars indicate the standard deviation of triplicate samples. (C and D) Same experiment: immunofluorescence assay (IFA) images of bat cell lines exposed to MERS-CoV/EMC (C) or MERS-CoV/Jor. (D) 1 (D1) or 3 (D3) days after virus exposure and stained against MERS-CoV spike protein (green). (E) Same experiment: TEM images of bat cells infected with MERS-CoV/EMC at day 1 after virus exposure. Red arrows point at double-membrane vesicles (DMVs) typical of coronavirus infections.

As bats could be a potential reservoir for MERS-CoV, we tested the susceptibility of ten diverse bat cells lines to infection with MERS-CoV/EMC or MERS-CoV/Jor at an MOI of 1. Viral titers in cell culture supernatants were determined by plaque assay on days 0, 1, 3, and 5 post-exposure of MERS-CoV. Six out of ten cell lines (EidNi/41.3, EpoNi/22.1, HypLu/45.1, HypNi/1.1, RoNi/7.1, and RoNi/7.2) propagated MERS-CoV (Figure 1A, 1B). In some cell lines (e.g., RoNi/7.2, EidNi/41.3) virus yields reached those observed in positive control (Vero E6) cells. PESU-B5L, R05T, R06E, and Tb1Lu cell lines did not support productive MERS-CoV virus infection (Figure 1A, 1B).

These results were also confirmed by immunofluorescence assay (IFA) in cell lines inoculated with MERS-CoV/EMC (Figure 1C) or MERS-CoV/Jor (Figure 1D). Infected cells were detected by MERS-CoV spike protein IFA. Representative images were picked from those taken on day 1 or day 3 post-exposure, as cytopathic viral effects diminished immunofluorescence in some cell lines (e.g., EidNi/41.3, RoNi/7.1, RoNi/7.2 cells, Figure 1C). Images of bat cells (day 1 post-exposure) inoculated with MERS-CoV/EMC taken by a transmission electron microscope (TEM, Figure 1E) show intracellularly budding virions in the endoplasmic reticulum-Golgi intermediate compartment (ERGIC), as well as double membrane vesicles (DMVs).

### Cell-surface expression of CD26/DPP4 on bat cells

**Figure 2:**
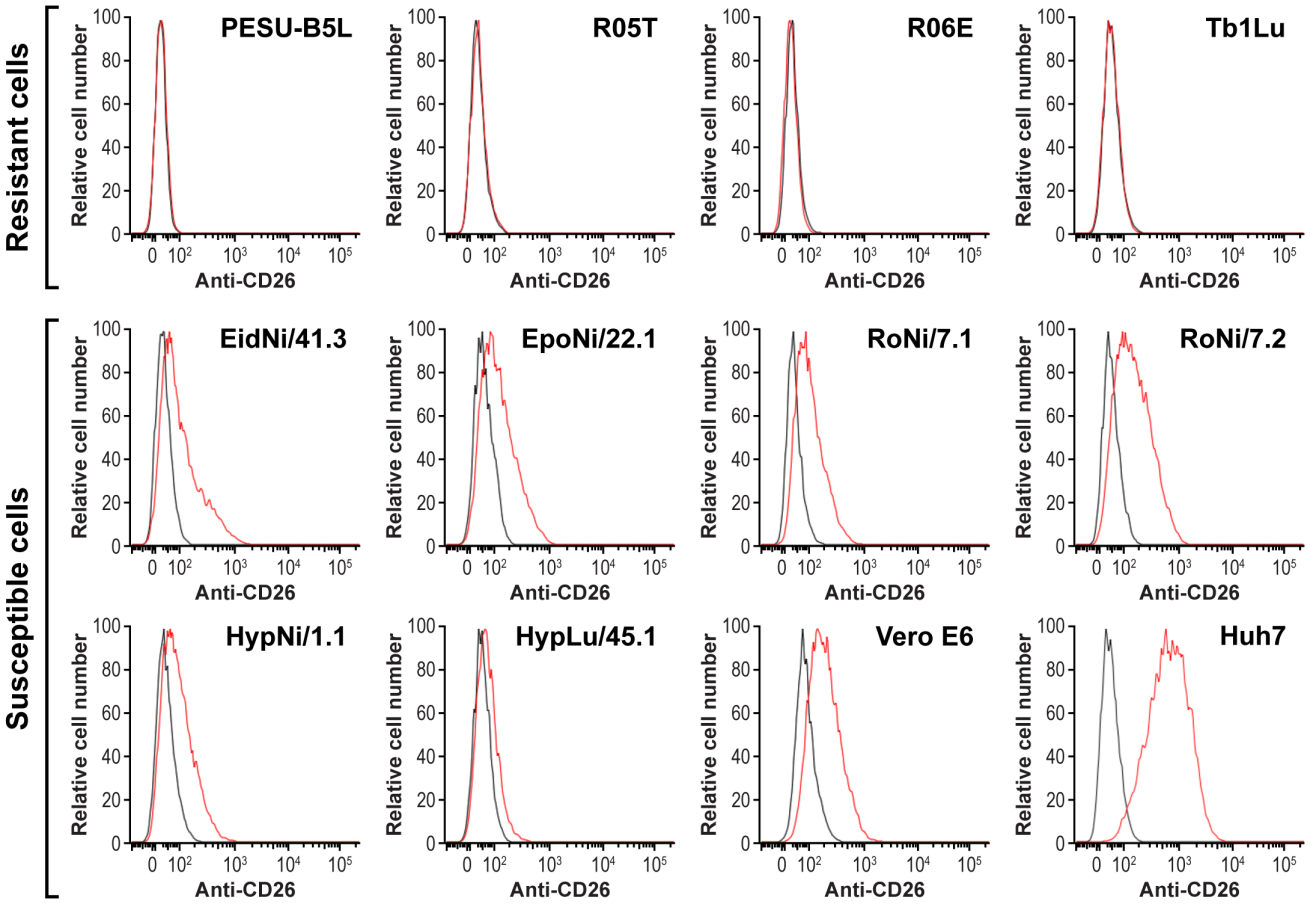
Cell-surface expression of CD26/DPP4 on bat cells. Cells of different bat cell lines were analyzed by flow cytometry after staining with goat anti-human DPP4/CD26 antibody (red line) or control antibody (black lines).

To evaluate whether CD26/DPP4 expression is related to susceptibility of bat cells to MERS-CoV infection, surface expression of CD26/DPP4 was analyzed in ten bat cell lines by flow cytometry using a polyclonal anti-human CD26/DPP4 antibody. None of the four MERS-CoV-resistant cell lines tested in this study (PESU-B5L, R05T, R06E, and Tb1Lu) were recognized by anti-human CD26/DPP4 antibody in this assay, whereas all susceptible bat cells (EidNi/41.3, EpoNi/22.1, HypLu/45.1, HypNi/1.1, RoNi/7.1, and RoNi/7.2) tested positive for CD26/DPP4 expression (Figure 2).

### Anti-human CD26/DPP4 antibody inhibits MERS-CoV infection in bat cells

**Figure 3:**
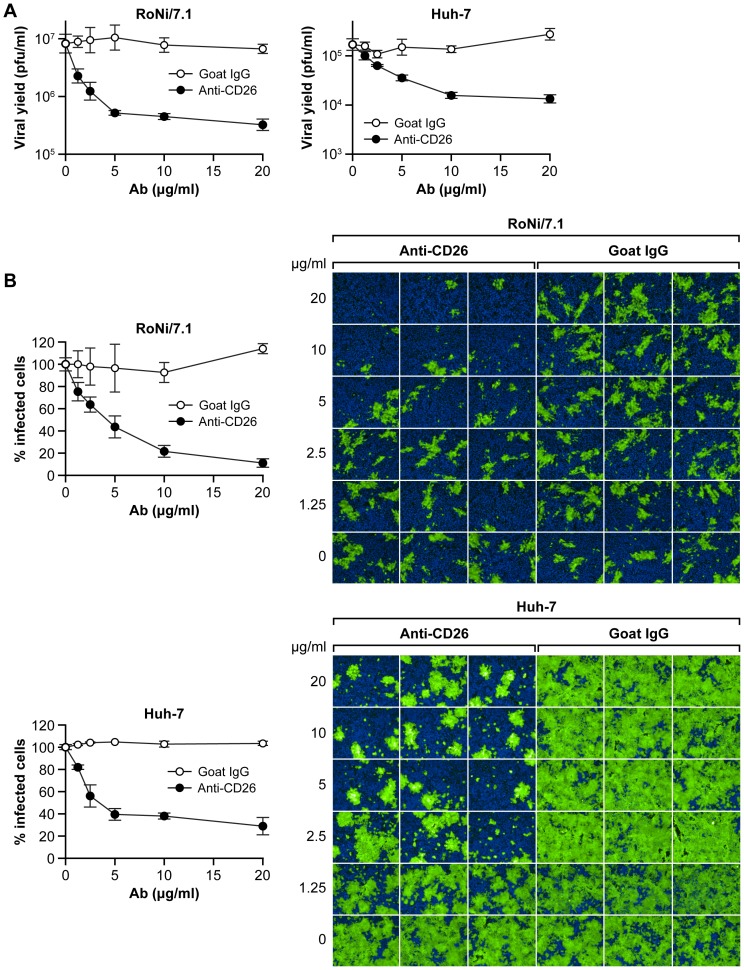
Anti-human CD26/DPP4 antibody inhibits MERS-CoV infection of bat cells. RoNi/7.1 or Huh-7 cells (control) were treated with increasing concentrations (0, 1.25, 2.5, 5, 10, and 20 µg/ml) of anti-human CD26/DPP4 antibody or control antibody and then exposed to MERS-CoV/EMC at an MOI of 1. (A) After 24 h, viral yields in supernatants were determined by plaque assay. (B) Cellular infection was determined by immunofluorescence assay (IFA) with an anti-MERS-CoV spike protein antibody (green). (B left) The percentage of infected cells was analyzed by high content imaging. (B right) Representative IFA images. Error bars indicate the standard deviation of triplicate samples.

To confirm the role of CD26/DPP4 in MERS-CoV bat cell entry, RoNi/7.1 cells or control human Huh-7 cells were incubated with increasing concentrations of a monoclonal anti-human CD26/DPP4 antibody and subsequently exposed to MERS-CoV/EMC. CD26/DPP4 antibody treatment reduced MERS-CoV particle production in both RoNi/7.1 cells and Huh-7 cells, as evidenced by a dose-dependent reduction in viral yield in plaque assays (Figure 3A). Immunofluorescent images of infected cells stained against MERS-CoV spike protein confirm these results (Figure 3B). Thus, CD26/DPP4 plays a crucial role in MERS-CoV cell entry into bat cells.

### Expression of human CD26/DPP4 confers MERS-CoV susceptibility to otherwise resistant bat cells

**Figure 4:**
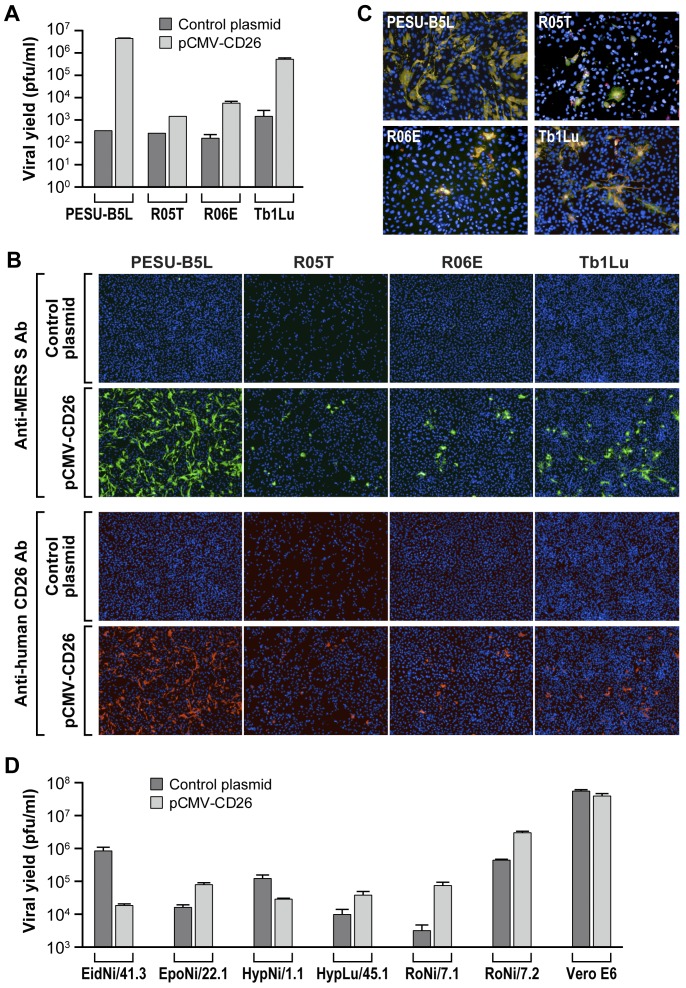
Expression of human CD26/DPP4 confers MERS-CoV susceptibility to otherwise resistant bat cells. (A) Viral yields from MERS-CoV-resistant PESU-B5L, R05T, R06E, and Tb1Lu bat cells. Cells were transfected with a plasmid expressing human CD26/DPP4 or empty control plasmid and exposed 48 h later to MERS-CoV/EMC at an MOI of 3. Supernatants were harvested at 24 h after virus exposure for quantification of virus yields by plaque assay. (B) Same experiment: representative immunofluorescence assay (IFA) images of cells stained with anti-MERS-CoV spike protein antibody (green, top) or anti-human CD26/DPP4 antibody (red, bottom). (C) Merged IFA images demonstrate colocalization of MERS-CoV spike protein and CD26/DPP4. (D). Viral yields from MERS-CoV-susceptible bat cells transfected with a plasmid expressing human CD26/DPP4 or empty control plasmid using procedures identical to resistant cells in (A) except that cells were exposed to virus 24 h after transfection. Error bars indicate the standard deviation of duplicate samples.

To test the hypothesis whether CD26/DPP4 alone determines bat cell susceptibility to MERS-CoV infection, we transfected MERS-CoV-resistant bat cell lines (PESU-B5L, R05T, R06E, TblLu) with a human CD26/DPP4 expression plasmid and then inoculated the cells with MERS-CoV. Transient expression of human CD26/DPP4 in these bat cell lines supported MERS-CoV replication as evidenced by increased viral yields compared to those measured in the same cell line transfected with empty control plasmid (Figure 4A). These results were confirmed by IFA using the same cells stained against MERS-CoV spike protein or CD26/DPP4 (Figure 4B). The merged immunofluorescent images clearly indicate the colocalization of MERS-CoV spike protein and CD26/DPP4 (Figure 4C). Thus, the restriction of MERS-CoV infection in the resistant bat cell lines may be determined by the absence of CD26/DPP4. Overexpression of human CD26/DPP4 in already susceptible bat cell lines led to an increase in virus yield in some bat cell lines (EpoNi/22.1, HypLu/45.1, RoNi/7.1, RoNi/7.2) or to a decrease in virus yield in other susceptible bat cell lines (EiD/41.3, HypNi/1.1, Figure 4D).

### Persistent MERS-CoV infection induces downregulation of bat cell CD26/DPP4 expression

**Figure 5:**
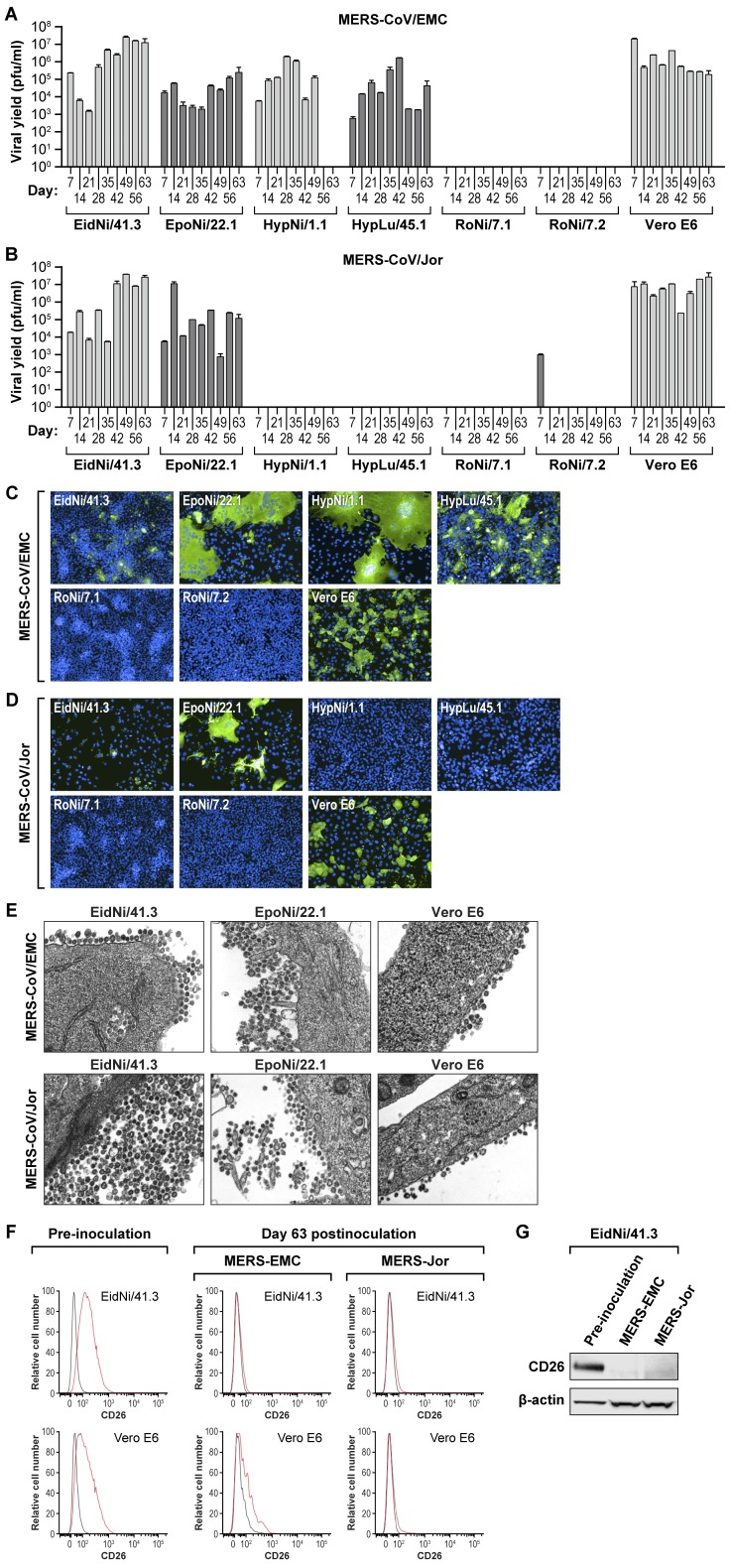
Persistent MERS-CoV infection of bat cells induces downregulation of bat cell CD26/DPP4 expression. Bat cell lines susceptible to infection were infected with MERS-CoV/EMC (A) or MERS-CoV/Jor (B) at an MOI of 1. After 7 days, supernatants were harvested for virus yield analysis by plaque assay, and the cells were subcultured at a 1∶10 dilution in new flasks. Subsequently, the persistently infected cells were passaged at a 1∶10 dilution weekly. Error bars indicate the standard deviation of duplicate samples. (C and D) Same experiment: immunofluorescence assay (IFA) images of bat cells persistently infected with MERS-CoV/EMC (C) or MERS-CoV/Jor (D) at day +33 stained with anti-MERS-CoV spike protein antibody (green). (E) Same experiment: TEM images of bat cells persistently infected with MERS-CoV/EMC at day 56. (F) Flow cytometry data of CD26/DPP4 surface expression (red line: anti-human CD26-/DPP4 antibody; black line: control antibody) in persistently infected cells. (G) CD26/DPP4 expression in persistently infected EidNi/41.3 cells (day 63) as detected by western blot.

Persistent subclinical infection in a mammalian host reservoir is a hallmark of numerous zoonotic viruses. To investigate whether MERS-CoV can establish persistent infections in bats on the cellular level, we infected susceptible bat cell lines (i.e., EidNi/41.3, EpoNi/22.1, HypLu/45.1, HypNi/1.1, RoNi/7.1, RoNi/7.2) with MERS-CoV/EMC or MERS-CoV/Jor, and serially passaged these cells weekly for a total of 9 weeks (63 days). Persistent infection, as indicated by viral yields determined by plaque assay, was achieved in EidNi/41.3, EpoNi/22.1, HypNi/1.1, and HypLu/45.1 cell lines, control Vero E6 cells inoculated with MERS-CoV/EMC (Figure 5A), and EidNi/41.3 and EpoNi/22.1 cell lines inoculated with MERS-CoV/Jor (Figure 5B). Immunofluorescent images taken on day 33 after staining against MERS-CoV spike protein also confirm persistent infection in the same cell lines (Figure 5C, 5D). Intracellular viral particles, virion budding, and virion egress from EidNi/41.3 and EpoNi/22.1 cells and control Vero E6 cells are clearly visible on TEM images acquired at day 56 post-exposure (Figure 5E).

Flow cytometry was used to determine CD26/DPP4 cell-surface expression in EidNi/41.3 and control Vero E6 cells before virus exposure and at study endpoint (day 63) by incubation with an antibody against human CD26/DPP4 (Figure 5F). Prior to exposure of EidNi/41.3 cells to MERS-CoV, CD26/DPP4 expression (red line) was higher than that observed for isotypic IgG control (black line), confirming CD26/DPP4 cell-surface expression as shown previously (Figure 2). At day 63, CD26/DPP4 expression was nearly absent in EidNi/41.3 cells. At the same time point in control Vero E6 cells, CD26/DPP4 expression was decreased but still detected in cells inoculated with MERS-CoV/EMC, whereas surface expression of CD26/DPP4 could barely be detected in cells inoculated with MERS-CoV/Jor. Western blot analysis of EidNi/41.3 cells persistently infected with either MERS-CoV/EMC or MERS-CoV/Jor (day 63) also demonstrated no or minimal expression of CD26/DPP4 (Figure 5G).

## Discussion

*In vitro* studies revealed that MERS-CoV can infect cell lines derived from nonhuman primates, civets, rabbits, goats, cows, sheep, chickens, and pigs, but not cell lines derived from cats, dogs, hamsters, or mice [22], [24], [25]. In this study, we explored the potential of bats to be a reservoir for MERS-CoV infection [13]–[15] by evaluating MERS-CoV infection in bat cell lines. We expanded the number of bat cell lines that are now known to be susceptible to MERS-CoV and identified several bat cell lines that are resistant to infection (Figure 1). These bat cell lines are derived from bats living in geographically disparate areas. Interestingly, bat cell lines that were susceptible to MERS-CoV infection (EidNi/41.3, EpoNi/22.1, HypLu/45.1, HypNi/1.1, RoNi/7.1, and RoNi/7.2) originated from bats found in Western Asia and Northern Africa. In these geographic areas, domestic and wild dromedaries can be found and natural human MERS-CoV infections are recorded (Table 1).

CD26/DPP4 is the cellular receptor for MERS-CoV [19]. This evolutionary conserved dimeric ectopeptidase is differentially expressed in various tissues but may not be the sole determinant for susceptibility at the organism level [25]. Importantly, cellular susceptibility to MERS-CoV infection not only depends on the expression of CD26/DPP4, but also on its sequence. For instance, five amino-acid variations in the MERS-CoV-binding domain of hamster, ferret, and mouse CD26/DPP4 compared to human CD26/DPP4 have been linked to the resistance of hamster, ferret, and mouse cell lines to MERS-CoV infection [22]. Our study confirms the role of CD26/DPP4 as receptor for two divergent MERS-CoV isolates and correlates its presence or absence on the surface of bat cells directly with bat cell susceptibility or resistance to productive MERS-CoV infection (Figures 2 and 3). Bat cells that tested negative for CD26/DPP4 expression by flow cytometry using the polyclonal anti-human CD26/DPP4 antibody may possibly express a CD26/DPP4 ortholog that is not recognized by this antibody. Although the cells were derived from bats of different geographic origins, they could be rendered permissive by ectopic expression of human CD26/DPP4 (Figure 4A–C).

Overexpression of human CD26/DPP4 in already MERS-CoV-susceptible bat cell lines led to an increase in virus yield in some bat cell lines and to a decrease in virus yield in others (Figure 4D). We hypothesize that this variation may be due to differences in the number of bat CD26/DPP4 molecules on the cell-surface of each bat cell. For instance, overexpression of human CD26/DPP4 in a bat cell line with naturally high bat CD26/DPP4 surface expression may not have an effect on virion entry efficiency since all virions already find enough binding partners in the untransfected cell. Vice versa, if a bat cell line expresses little CD26/DPP4, finding a binding partner would present a bottleneck for MERS-CoV virions, and overexpression of human CD26/DPP4 might overcome this bottleneck and thereby increase virus yield. Second, we hypothesize that overexpression of human CD26/DPP4 may interfere with transport and/or functionality of certain bat CD26/DPP4 orthologs due to heterodimerization and consequent structural changes.

Our results indicate that, at least on the cellular level, presence or absence of functional CD26/DPP4 with a suitable MERS-CoV-binding domain is a major determinant for MERS-CoV cellular tropism in bats. In addition to potential differences in the MERS-CoV-binding domain of distinct bat CD26/DPP4 orthologs, presence or absence of yet-to-be-identified co-receptors and bat species-specific cellular factors acting downstream of virion adsorption and fusion may further influence to what extent a productive infection can be established. However, these considerations alone are not sufficient to pinpoint bats as epidemiologically relevant MERS-CoV hosts, as the immune system at the level of the organism may interfere with infection prior to cell entry or lead to rapid viral clearance.

One characteristic of natural virus host reservoirs is that hosts frequently are persistently infected with virus in the absence of clinical signs. Continuous transmission of viruses from such a reservoir to other animals, including humans, is a hallmark of zoonoses and explains repeated introduction of viruses into susceptible animal populations [26]. A number of zoonotic viruses are known to persistently and subclinically infect rodents (e.g., arenaviruses, hantaviruses) or bats (henipaviruses), which then directly or indirectly infect humans [17], [27]–[31]. Among the betacoronaviruses, the group of coronaviruses including MERS-CoV, murine coronavirus and severe acute respiratory syndrome (SARS)-related coronaviruses that are related to MERS-CoV, are known to cause persistent infection in their mammalian hosts [16], [32]. During persistent SARS-CoV infection in Vero E6 cells and also during acute infection in laboratory mice, the SARS-CoV entry receptor, angiotensin converting enzyme 2 (ACE2), is downregulated [33], [34]. ACE2 downregulation contributes to the severity of lung pathology of SARS [34].

In this study, we established persistent infection (up to 63 days post-exposure) in EidNi/41.3, EpoNi/22.1, HypNi/1.1, and HypLu/45.1 bat and control Vero E6 cells using MERS-CoV/EMC, and in EidNi/41.3 and EpoNi/22.1 bat and control Vero E6 cells using MERS-CoV/Jor. Interestingly, sequencing of the receptor-binding domain of MERS-CoV/EMC- and MERS-CoV/Jor-containing samples taken on day 63 from persistently infected EiDNi/41.3 and Vero E6 cells did not reveal any amino-acid changes compared to the wild-type/reference sequences (GenBank: JX869059.2 and KC776174.1, respectively; data not shown). Similar to SARS-CoV-induced ACE2 downregulation, persistent MERS-CoV infection in bat cell lines and Vero E6 cells was associated with downregulation of CD26/DPP4 (Figure 5). The observation that cultures continued to release significant virion amounts on day 63 post-inoculation, but also exhibited low to absent CD26/DPP4 cell-surface expression, is consistent with receptor downregulation as one mechanism for persistent infection with MERS-CoV in our experiments. Virus carryover between cell/virus passages probably did not influence these results as excreted viruses should be unable to re-infect cells in the absence of CD26/DPP4 receptor. The mechanism of CD26/DPP4 downregulation upon persistent MERS-CoV infection requires further study. Western blot-based examination of lysates of persistently infected cells at day 63 indicate that CD26/DPP4 is either not expressed anymore or is very efficiently degraded (Figure 5G).

The reason for the observed differences in establishing persistent infection in these cell lines among the two different MERS-CoV isolates is unclear, but may have epizootiological significance and needs to be examined in future studies.

Our results are consistent with the suggested role of bats in MERS-CoV transmission. If CD26/DPP4-positive cells of bats become infected with MERS-CoV and if subsequently the receptor surface expression is downregulated as we observe in our experiments, a persistent infection could be established. Such animals may provide a reservoir that continuously sheds infectious virus, possibly transmitting the virus to other mammals, such as dromedaries. Further evaluation is needed to determine whether downregulation of CD26/DPP4 is a general hallmark of persistent MERS-CoV infection in animal models of MERS and whether the receptor influences pathogenesis in a manner similar to that seen with ACE2 downregulation in persistent SARS-CoV infection.
